# Association between anxiety and skin conductance according to the intensity of shaking of virtual reality images

**DOI:** 10.3389/fpsyt.2023.1196767

**Published:** 2023-10-03

**Authors:** Dong Jun Kim, Hyewon Kim, Kiwon Kim, Min-Ji Kim, Hong Jin Jeon

**Affiliations:** ^1^Samsung Medical Center, Sungkyunkwan University, Seoul, Republic of Korea; ^2^Meditrix Co., Ltd., Seoul, Republic of Korea; ^3^Department of Health Sciences and Technology, Samsung Advanced Institute for Health Sciences & Technology (SAIHST), Sungkyunkwan University, Seoul, Republic of Korea; ^4^Department of Psychiatry, Kangdong Sacred Heart Hospital, Hallym University College of Medicine, Seoul, Republic of Korea; ^5^Biomedical Statistics Center, Samsung Medical Center, Research Institute for Future Medicine, Seoul, Republic of Korea; ^6^Department of Health Sciences and Technology, Medical Device Management and Research, Clinical Research Design and Evaluation, SAIHST, Sungkyunkwan University, Seoul, Republic of Korea

**Keywords:** virtual reality, anxiety, skin conductance, cyber sickness, depression

## Abstract

**Introduction:**

Despite the advantages of virtual reality (VR), cyber sickness makes it difficult to apply VR to those who are already anxious and in distress. Skin conductance (SC) is widely used as a bio-signal reflecting anxiety. It is positively correlated with anxiety. The objective of this study was to determine the association between SC and anxiety in VR.

**Methods:**

Healthy volunteers with moderate-to-high stress defined as a Perceived Stress Scale-10 (PSS-10) score ≥20 were enrolled. STAI-X-1 was used to measure anxiety, and galvanic skin response was used to measure SC. This study used an open, randomized, crossover design. In this study, 360° videos consisted of two types, namely, less dizzying video (G1) and more dizzying video (G2). We randomized subjects into two groups according to video exposure order: G1 after watching G2 (Order 1) and G2 after watching G1 (Order 2). Of 81 subjects, the average age (±SD) was 39.98 ± 10.94 years for the Order 1 group and 36.54 ± 12.44 years for the Order 2 group.

**Results:**

Anxiety was significantly decreased in the Order 2 group (*p* < 0.035) after watching videos, whereas there was no significant change in anxiety in the Order 1 group. In both groups, SC was significantly increased after exposure to a dizzying video. Mean difference (SD) between the second VR video and baseline SC was 1.61 (1.07) (*p* < 0.0001) in the Order 1 group and 0.92 (0.90) (*p* < 0.0001) in the Order 2 group, showing a significant difference between the two groups (*p* < 0.003). However, there was no significant difference between the two groups (*p* < 0.077) after baseline correction.

**Conclusion:**

Anxiety was decreased significantly in the Order 2 group. The Order 1 group showed a high rate of change in skin conductivity. It is possible to reduce SC and anxiety by viewing a less dizzying VR video first and then viewing a more dizzying video later.

## 1. Introduction

Virtual reality (VR) is being used in various fields of medicine along with the development of head-mounted display (HMD) devices (1–3). In psychiatry, VR has been mainly used to treat patients with specific phobia or posttraumatic stress disorder (PTSD) (4–8). Recently, VR has been widely used to treat various other mental disorders (9–11). However, since VR can cause cyber sickness, caution is needed to use VR for therapeutic intervention (12). Cyber sickness is a collection of symptoms that occur during exposure to VR immersion, including eye strain, headache, vertigo, ataxia, sweat, disorientation, and dizziness (13–15). To improve treatment efficacy for patients with anxiety, it is necessary to investigate the increase in anxiety caused by cyber sickness when watching VR videos.

Skin conductance (SC) is widely used as a bio-signal reflecting anxiety because it is positively correlated with anxiety (16–18). SC, also referred to as electrodermal activity (EDA), is the measurement of the electrical conductivity of the skin (19). It has been found that depression is associated with a decrease in EDA (20), while fear and anxiety are associated with an increase in EDA (21). The EDA level (tensile level of electrical conductivity) is measured by placing two electrodes in a dense position of the eccrine sweat gland. It is typically measured for fingers or hands (22). However, there are variations depending on the user's skin condition. A high value is measured in the case of a sweaty constitution, while a low value is measured in the case of severe dry skin.

Based on the fact that shaking of the video causes cyber sickness (13–15), the purpose of this study was to determine whether the order of exposure to the VR image with increasing or decreasing the degree of dizziness was associated with participant's anxiety and whether such anxiety could be measured in real time based on skin conductance. The association between anxiety and skin conductance was analyzed by varying the exposure order of VR images. In treatment with virtual reality, it will be important to know whether the most dizzy substance should be introduced at the end of treatment or at the beginning of treatment to reduce cyber sickness. This study can help solve the problem. We hypothesized that exposure to dizzy images would increase anxiety and skin conductivity compared to baseline.

## 2. Methods

### 2.1. Participants

This study used an open, randomized, crossover design and a secondary analysis study in the same data set as described in a previous study (23). The order of exposure to dizziness was randomized and blinded. Among 83 participants, two with missing data were excluded from the analysis. A total of 81 healthy volunteers with moderate-to-high stress defined as a Perceived Stress Scale-10 (PSS-10) score ≥20 (24) were analyzed. Participants with major depressive disorder, bipolar disorder, schizophrenia, other psychotic disorders, delusional disorder, anxiety disorder, delirium, dementia, eating disorder, alcohol use disorders, organic mental disorders, intellectual disorder, psychiatric disorders due to other medical conditions, suicidal risk, neurological illnesses including stroke or epilepsy, or serious medical illnesses were excluded. Those with a medical or surgical history of otorhinolaryngologic, ophthalmologic disorders, or problems with neck movements were also excluded. A psychologist who was specialized in this psychiatric evaluation administered the Korean version of Mini-International Neuropsychiatric Interview (MINI) (25) according to the Diagnostic and Statistical Manual of Mental Disorders (DSM-5) (26) to subjects to evaluate psychiatric disorders. This study was approved by the Institutional Review Board (IRB) of Samsung Medical Center (IRB No. SMC 2016-10-007-004). All participants gave written informed consent upon enrollment for this study. They were provided financial compensation for participating in this study.

### 2.2. Procedure

**Figure 1:**
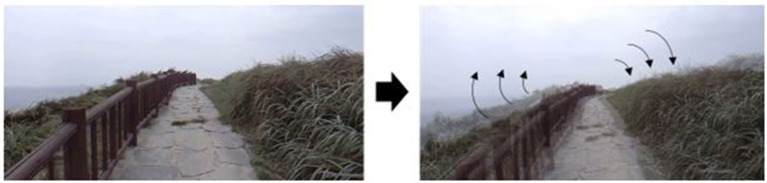
Dizzy 360° virtual reality video. The original image **(left)** provided by the Ministry of Land was measured to be 0.004°/sec. An image with 0.3°/sec (Grade1) and 0.38°/sec (Grade2) was created by artificially adding a Sin waveform roll rotation shaking of 30 Hz in the Z-axis direction.

The original 360° video (Jeju Island scene) was provided by the Korea Land and Geospatial InformatiX Corporation. Along with Samsung Electronics, it was modified to be a second stage of dizzy video. Dizzy videos were artificially modified by adding a rolling swing of a sine waveform of 30 Hz in the Z-axis direction with 0.008°/s for each grade based on previous studies (27–29). We then made an image movement of 0.3°/s (G1) and 0.38°/s (G2) (Figure 1). Videos consisted of two types, namely a less dizzying video (G1) and a more dizzying video (G2). We randomized subjects to two groups according to exposure order, G1 after watching G2 (Order 1) and G2 after watching G1 (Order 2).

This study was conducted with two visits at intervals of 2 weeks. It was conducted in the morning and afternoon to check virtual reality adaptation. Our research study was conducted in a room in the Clinical Trial Center of Samsung Medical Center that could completely block out the noise outside. We used Samsung Gear VR Head Mounted Device (HMD) with Galaxy S7 Smartphone. Participants were exposed to dizzy VR video for a total of 7 min.

### 2.3. Measures

Galvanic skin response (GSR) was used to measure skin conductance. GSR sensors were attached to the second and fourth fingers for measurement (30, 31) using a computerized biofeedback system, ProComp Infiniti (Thought Technology Ltd., Montreal, Canada) (32). Association with SC and anxiety after watching dizzy videos was investigated using the STAI-X-1, a representative anxiety evaluation tool (33). The STAI-X-1 is a self-report consisting of 20 items for measuring the anxiety level. Its scale scores ranged from 20 to 80, with a higher score indicating a higher level of anxiety. Participant's characteristics such as height, weight, body mass index (BMI), blood pressure, pulse rate, and body temperature were also collected.

### 2.4. Statistical analysis

Continuous variables are expressed as mean ± standard deviation (SD). Categorical variables are presented as number (percentage). Comparisons between two order groups were conducted with the chi-square test or Fisher's exact test for categorical parameters and the *t*-test or Wilcoxon rank-sum test for continuous parameters, as appropriate. Changes among three time points (baseline, first VR, second VR) were defined by the difference between the previous time point and the later time point. Change rate was calculated as the change divided by the baseline value. The significance of a change in each group was determined by the paired *t-*test or Wilcoxon signed rank test. After adjusting for day (day 1 or day 2) and time (am or pm), comparison of change rate between the two order groups was conducted using generalized estimating equations (GEE). Statistical significance was considered at a *P-*value of < 0.05. All statistical analyses were performed using SAS version 9.4 (SAS Institute Inc., Cary, NC, USA).

## 3. Results

Demographic characteristics were not significantly different between the two groups except for pulse rate (*p* < 0.027). As shown in Table 1, mean age was 39.98 years for the Order 1 group and 36.54 years (*p* < 0.205) for the Order 2 group. In the Order 1 group, 45% were male patients and 55% were female patients. In the Order 2 group, 51.22% were male patients and 48.78% were female patients (*p* < 0.575). The percentage of those without motion sickness was 72.5% in the Order 1 group and 85.37% in the Order 2 group (*p* < 0.155).

**Table 1:** Comparison of baseline characteristics of subjects in the two order groups.

		**Order 1: G2 → G1 (*n* = 40)**	**Order 2: G1 → G2 (*n* = 41)**	***p*-value**
Age (Year)		39.98 ± 10.94	36.54 ± 12.44	0.2053
Sex	Male	18 (45)	21 (51.22)	0.5754
	Female	22 (55)	20 (48.78)	.
Motion sickness	No	29 (72.5)	35 (85.37)	0.1551
	Yes	11 (27.5)	6 (14.63)	.
Migraine	No	35 (87.5)	35 (85.37)	0.7792
	Yes	5 (12.5)	6 (14.63)	.
Education (Year)		15.15 ± 2.25	14.73 ± 3.07	0.4886
Height (cm)		165.03 ± 7.52	166.54 ± 12.1	0.2000
Weight (kg)		63.05 ± 10.85	67.49 ± 13.21	0.1537
BMI (kg/m^2^)		22.5 ± 3.08	23.46 ± 3.47	0.1907
Systolic blood pressure (mmHg)		125.5 ± 14.25	125.2 ± 15.71	0.9274
Diastolic blood pressure (mmHg)		74.5 ± 11.4	74.02 ± 13.76	0.8661
Pulse rate		87.83 ± 13.23	81.39 ± 12.39	**0.0266**
Body temperature (°C)		36.43 ± 0.36	36.36 ± 0.3	0.4989
Alcohol	No	15 (37.5)	19 (46.34)	.
	Yes	25 (62.5)	22 (53.66)	.
	Frequency	1.32 ± 0.63	1.45 ± 0.80	0.5679
Smoking history	No	29 (72.5)	34 (82.93)	0.3453
	Yes	10 (25)	7 (17.07)	.
	Ex-smoker	1 (2.5)		.

Data are presented as mean ± standard deviation or *n* (%).

*P*-values were obtained by the chi-square test or Fisher's exact test for categorical variables and the *t*-test or Wilcoxon rank-sum test for continuous variables. BMI, body mass index; Ex-smoker, electronic cigarette; G1, Grade1 = less dizzying video; G2, Grade2 = more dizzying video. Bold values indicate *p*-value of 0.05 or less.

As shown in Table 2, SC was increased significantly in both groups after VR video exposure than that at baseline (*p* < 0.0001). Mean (±SD) difference of SC between the first VR and the baseline was 1.54 (±1.17) for the Order 1 group and 0.85 (±0.86) for the Order 2 group. Changes in SC in the two groups between the first VR and the baseline were statistically significant (*p* < 0.005). Mean (±SD) difference of SC between the second VR and the baseline was 1.61 (±1.07) for the Order 1 group and 0.92 (±0.9) for the Order 2 group. Changes in SC in the two groups between the second VR and the baseline were statistically significant (*p* < 0.003). The difference (mean ± SD) of SC between the second VR and the first VR in the Order 1 group was 0.07 ± 0.47, which was statistically significant (*p* < 0.036). STAI-X-1 was decreased significantly after the first VR exposure (*p* < 0.001) and the second VR exposure (p < 0.035) as compared to baseline in the Order 2 group. Mean (±SD) change of STAI-X-1 in the Order 2 group was −4.24 (±6.66) between the first VR and baseline and −3.12 (±9.16) between the second VR and baseline. Changes in STAI-X-1 between the first VR and the baseline were statistically significant between the two groups (*p* < 0.031).

**Table 2:** Significance of changes in skin conductance and STAI-X-1 at day 1 AM in each order group.

			**Order 1: G2**→**G1**		**Order 2: G1**→**G2**		**Order 1 vs. order 2**
			**Mean** ±**SD**	***p*** **-value**	**Mean** ±**SD**	***p*** **-value**	***p*** **-value**
STAI-X-1	Measurement at each time point	Baseline	48.75 ± 8.47		45.68 ± 11.06		
		After first VR	48.2 ± 10.66		41.44 ± 11.12		
		After second VR	49.1 ± 11.63		42.56 ± 11.96		
	Change	First VR-baseline	−0.55 ± 8.39	0.6809	−4.24 ± 6.66	**0.0002**	**0.0310**
		Second VR-1st VR	0.9 ± 3.89	0.1512	1.12 ± 4.73	0.1370	0.8185
		Second VR-baseline	0.35 ± 9.41	0.8153	−3.12 ± 9.16	**0.0350**	0.0963
Skin conductance (μS)	Measurement at each time point	Baseline	0.65 ± 0.61		0.48 ± 0.58		
		After first VR	2.19 ± 1.65		1.33 ± 1.3		
		After second VR	2.26 ± 1.5		1.41 ± 1.36		
	Change	First VR-baseline	1.54 ± 1.17	**<0.0001**	0.85 ± 0.86	**<0.0001**	**0.0044**
		Second VR-1st VR	0.07 ± 0.47	**0.0355**	0.07 ± 0.22	0.0584	0.2177
		Second VR-baseline	1.61 ± 1.07	**<0.0001**	0.92 ± 0.9	**<0.0001**	**0.0029**

*P*-values were obtained by the paired *t*-test or Wilcoxon signed rank test in each group and the *t*-test or Wilcoxon rank-sum test for comparison of groups; STAI, state-trait anxiety inventory; G1, Grade1 = less dizzying video; G2, Grade2 = more dizzying video; SD, standard deviation; VR, virtual reality video. Bold values indicate *p*-value of 0.05 or less.

As shown in Table 3, skin conductance difference after baseline correction was not significantly different between the two groups (*p* >0.05). Mean (± SD) difference of SC for Day 1 PM between the second VR and baseline was 3.23 (±3.6) for the Order 1 group and 2.2 (±2.41) for the Order 2 group.

**Table 3:** Comparison of change rate in skin conductance between the two order groups.

		**Order 1: G2 → G1 (*n* = 40)**	**Order 2: G1 → G2 (*n* = 41)**	***p*-value**
**Time**	**The change rate**	**Mean** ±**SD**	**Mean** ±**SD**	
Day 1-AM	First VR-baseline	3.22 ± 3.17	2.13 ± 2.02	0.0814
	Second VR-first VR	0.11 ± 0.24	0.12 ± 0.3	0.6811
	Second VR-baseline	3.52 ± 3.34	2.37 ± 2.07	0.0765
Day 1-PM	First VR-baseline	2.41 ± 2.75	1.76 ± 2.08	0.0719
	Second VR-first VR	0.27 ± 0.33	0.18 ± 0.31	0.0595
	Second VR-baseline	3.23 ± 3.6	2.2 ± 2.41	0.0500
Day 2-AM	First VR-baseline	1.46 ± 1.56	1.33 ± 1.51	0.2396
	Second VR-first VR	0.21 ± 0.33	0.15 ± 0.23	0.7660
	Second VR-baseline	1.93 ± 1.92	1.68 ± 1.93	0.1579
Day 2-PM	First VR-baseline	1.1 ± 0.75	1.22 ± 1.62	0.1343
	Second VR-first VR	0.32 ± 0.48	0.2 ± 0.24	0.1393
	Second VR-baseline	1.79 ± 1.61	1.74 ± 2.22	0.0993
Overall	First VR-baseline	2.05 ± 2.40	1.61 ± 1.84	0.2049
	Second VR-first VR	0.23 ± 0.36	0.16 ± 0.27	0.1308
	Second VR-baseline	2.62 ± 2.83	2.00 ± 2.16	0.1496

The change rate was calculated as the change divided by the baseline value. *P*-values were obtained by the Wilcoxon rank-sum test or analysis using GEE adjusted by day and time. *P*-values by the Wilcoxon rank-sum test at each time point and analysis using GEE adjusted by day and time for repeated measurements; G1, Grade1 = less dizzying video; G2, Grade2 = more dizzying video; SD, standard deviation; VR, virtual reality video.

**Figure 2:**
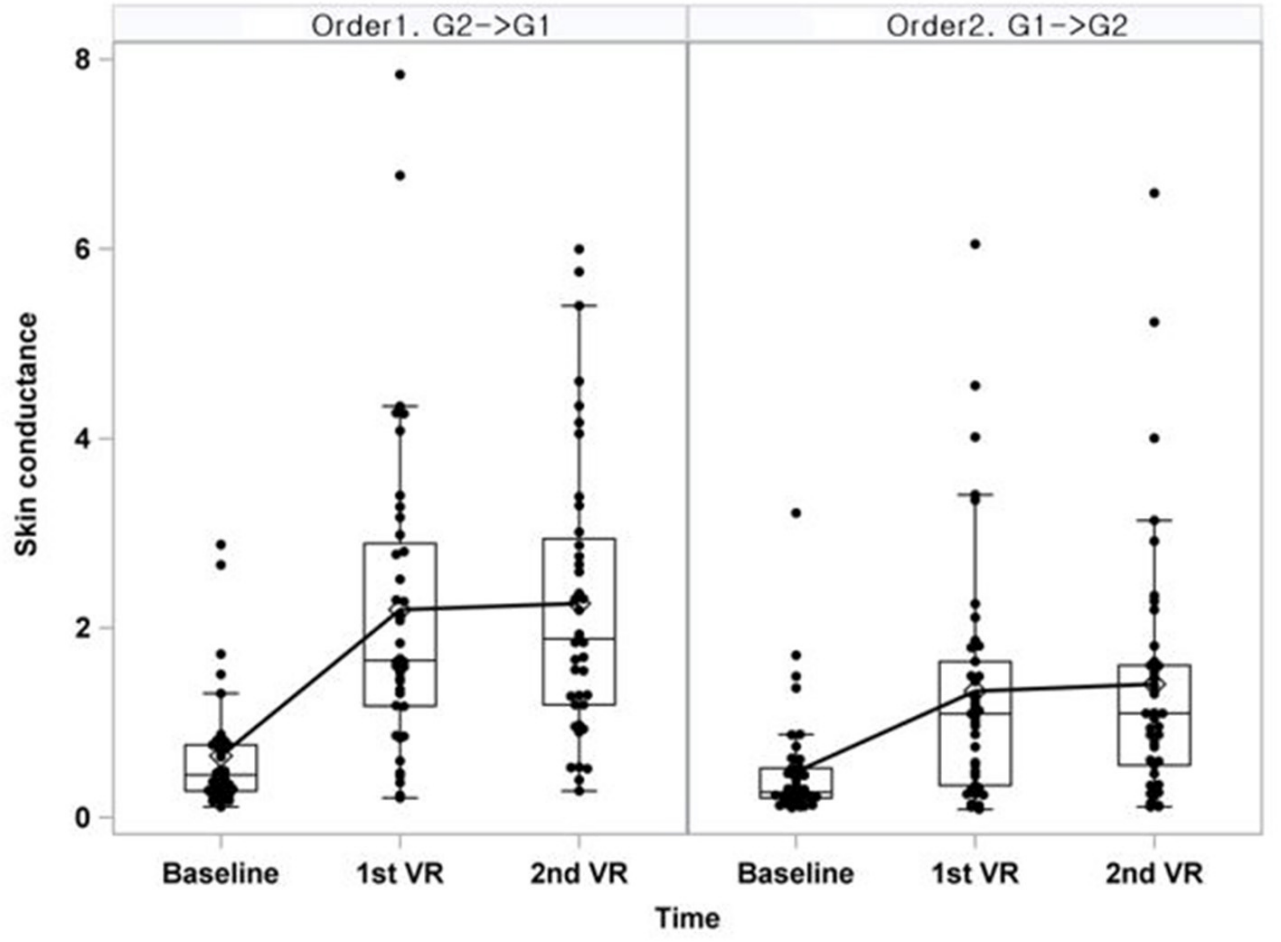
Box-whisker plot of skin conductance at day 1-AM. Line was connected with the mean of each time point; G1, Grade1 = less dizzying video; G2, Grade2 = more dizzying video; SD, standard deviation; VR, virtual reality video.

All measurements of skin conductance by time point and the line connecting the average time point are displayed in a box-whisker plot (Figure 2). Skin conductance was found to be steeper in the group that watched more dizzy VR than that in the group that watched less dizzy VR (Order 2) in the first virtual reality stimulus. Measurement of skin conductance was flat regardless of the intensity of dizziness in the second VR stimulus.

## 4. Discussion

We predicted that anxiety would gradually increase after exposure to a dizzy video compared to that at baseline. However, the results were somewhat different from our prediction. It might be due to the following two reasons. First, baseline anxiety was highly measured in both groups due to tension caused by the first experience of experimental environment. The second reason was that the dizzy video produced was based on natural scenery images. Thus, anxiety was measured to be lower than expected. In addition, the VR image was used based on what could be used to treat patients with high anxiety and minimize the burden on participants. However, it was meaningful that anxiety was slightly increased in the Order 1 group after the last video exposure, while anxiety was decreased statistically significantly in the Order 2 group (*p* < 0.035). This was in line with an increase in higher skin conductivity change rate in the Order 1 group after exposure to the last video (mean ± SD of SC difference between second VR and baseline: 1.61 ± 1.07). It could be interpreted that the increase in anxiety was greater when watching less dizzy videos after watching strong dizziness videos than in the opposite case.

The amount of change in skin conductance between the two groups did not show a significant difference after correcting the baseline. This might be due to the following reasons. First, pulse rate of the Order 1 group was significantly (*p* < 0.027) higher than that of the Order 2 group. This might have led to highly measured anxiety and skin conductance at baseline. Second, the number of participants was small. Skin conductance reacts sensitively to a sweaty constitution. If the skin is severely dry, there is a deviation such as a low measurement value. If the sample size is small, the deviation might be greater (21, 34). As shown in Table 3, from Day 1 AM to Day 2 PM, the average change of SC between first VR and baseline decreased from 3.22 to 1.1 in the Order 1 group and from 2.13 to 1.22 in the Order 2 group, showing a tendency to decrease. However, when the date and time were corrected, the difference between the two groups was insignificant (*p* = 0.2049). This indicates that when exposed to a more dizzy VR for the first time, the measurement of skin conductivity increases. When one gets used to VR later, there is no significant difference in the measurement regardless of which VR is watched first.

In conclusion, our study showed that anxiety and skin conductivity increased more when watching less dizzy video after watching a more dizzy video than in opposite situations. This might help us prepare image composition when VR is used for treatment intervention to reduce anxiety in future. It is possible to reduce skin conductance and anxiety by viewing less dizzying VR videos first and then viewing more dizzying videos later.

VR with HMD has the advantage of maximizing immersion. However, using 360° images has the disadvantage of causing cyber sickness depending on the degree of stabilization of the camera (35). In our study, it was confirmed that skin conductance was significantly increased after watching the last VR video. Since cyber sickness can cause headache, vomiting, and dizziness, attention should be paid to treatment intervention (36, 37). Therefore, when virtual reality is used for therapeutic intervention, a careful approach is needed. Previous studies have shown that breathing training is effective in reducing dizziness (38). In addition, considering the order of exposure to dizziness, it will be possible to make more effective settings when using virtual reality for therapeutic intervention. A more complete setting such as preparing images and environments to minimize cyber sickness is needed when VR is used for relaxation training of patients with depression and anxiety in future. However, caution is needed when generalizing the results of this study as this study was conducted only with Korean people. More studies enrolling subjects from various cultures are needed to verify our results.

## Data availability statement

The original contributions presented in the study are included in the article/supplementary material, further inquiries can be directed to the corresponding author.

## Ethics statement

The studies involving humans were approved by Institutional Review Board of Samsung Medical Center. The studies were conducted in accordance with the local legislation and institutional requirements. The participants provided their written informed consent to participate in this study.

## Author contributions

HK, KK, and HJ contributed to conception and design of the study. M-JK performed the statistical analysis. DK wrote the first draft of the manuscript. All authors contributed to manuscript revision, read, and approved the submitted version.
